# Contact pathway of coagulation and inflammation

**DOI:** 10.1186/s12959-015-0048-y

**Published:** 2015-05-06

**Authors:** Yi Wu

**Affiliations:** The Sol Sherry Thrombosis Research Center, Temple University School of Medicine, 3420 N. Broad Street, Philadelphia, PA 19140 USA

**Keywords:** Contact system, Coagulation, Inflammation, Platelet, Infection, Autoimmune disease, Vascular biology

## Abstract

The contact system, also named as plasma kallikrein-kinin system, consists of three serine proteinases: coagulation factors XII (FXII) and XI (FXI), and plasma prekallikrein (PK), and the nonenzymatic cofactor high molecular weight kininogen (HK). This system has been investigated actively for more than 50 years. The components of this system and their interactions have been elucidated from in vitro experiments, which indicates that this system is prothrombotic by activating intrinsic pathway, and proinflammatory by producing bioactive peptide bradykinin. Although the activation of the contact system have been implicated in various types of human disease, in only a few instances is its role clearly defined. In the last 10 years, our understanding of the contact system, particularly its biology and (patho)physiology has greatly increased through investigations using gene-modified animal models. In this review we will describe a revitalized view of the contact system as a critical (patho)physiologic mediator of coagulation and inflammation.

The contact system is a group of plasma proteins that responds to the presence of (patho)physiological materials and invasion pathogens. This system consists of three serine proteinases: coagulation factors XII (FXII) and XI (FXI), and plasma prekallikrein (PK), and the nonenzymatic cofactor high molecular weight kininogen (HK) [1]. These plasma proteins were grouped together as the contact system, because they required contact with artificial, negatively charged surfaces for zymogen activation in vitro. Upon the activation of this system, it can trigger blood coagulation and is responsible for the generation of the proinflammatory products such as bradykinin. This system is also called as plasma kallikrein-kinin system, functioning in a few inflammatory conditions such as rheumatoid arthritis (RA) and inflammatory bowel disease (IBD) [2].

Investigations of their biochemical and biologic properties have shown that the contact system proteins interact with a number of physiologic and pathophysiologic systems. The biological role of contact system is to initiate and participate in the pathophysiological responses to injury, mainly in the processes of coagulation and inflammation. The activation of this system has been shown to be involved in a wide variety of human disease states, including septicemia and endotoxemia, ARDS, DIC, typhoid fever, Rocky Mountain spotted fever, Crohn’s disease, transfusion reactions, renal allograft rejection, nephrotic syndrome, hereditary angioedema, and in extracorporeal circulation [1]. In animal models of sepsis, arthritis, and enterocolitis, the inhibitors of contact factor enzymes have modulated hypotension, inflammation or prolonged survival, suggesting the participation of this system in the host defense and innate immunity [3]. The contact system is evolutionarily conserved (deficiencies in contact factors are very rare), and the activation of the contact system have been implicated in various types of human disease, however, it currently still remains a mystery what its physiological function is in human. In the last 10 years, because of the studies using gene-modified animal models, our understanding of the contact system, particularly its physiology and pathophysiology, has greatly increased. In this review, we will first describe the molecular and cellular mechanisms that drive contact activation on nonphysiological materials. Next, we will summarize the clues linking the contact system with coagulation and inflammation in health and disease, and will discuss recent findings from both fundamental and clinical studies on the contributions of contact system to cardiovascular, infectious, and inflammatory and autoimmune disease, and their potential to be target for treatment of both thrombotic and inflammatory diseases.

## Assembly-based activation of contact system

In plasma, about 75% of PK circulates bound noncovalently to HK in 1:1 ratio, while the rest PK circulates freely. Besides PK, HK forms a heterodimer with factor XI, both of PK and FXI are surface bound via HK and share a conserved HK-binding site. The nonenzymatic cofactor HK is responsible for binding to negatively charged surfaces and assembly of these proteins forming a complex [1]. Once FXII is bound to surfaces, it undergoes conformational changes to become autoactivated (auto-activation), the activated FXIIa catalyzes the activation of PK. Meanwhile, FXII is increasingly susceptible to cleavage by plasma kallikrein (PK) and activated FXII (FXIIa) itself. Activated plasma kallikrein converts FXII into FXIIa (trans-activation), thereby forming an activation feedback loop, which overcomes inactivation of kallikrein and FXII by their counterpart inhibitors, such as C1-esterase inhibitor. In the absence of factor XII, prekallikrein does not become activated on an artificial surface. It is because of this finding that this system is called the contact system. In the early phase of contact activation, the independent binding ability of HK to the surface is limited. However, once PK and FXII are activated, single chain HK can be cleaved into two-chain HKa and release bradykinin. Compared with HK, HKa has greater capacity of binding to the surfaces and pro-inflammatory effect [4]. Moreover, Factor XI is activated in complex with HK and FXII, whereby the intrinsic pathway of coagulation is initiated. All these above information is accumulated from in vitro observations, how the contact system is activated in vivo has not been well-characterized.

### Molecules-mediated assembly and activation of contact system

The contact system is mostly known for its activation by negatively charged molecular surfaces, such as kaolin, glass, silica, ellagic acid, dextran sulfate, oversulfated chondroitin sulfate and nanoparticles [1]. Although these are multiple non-physiological contact activators, this reaction forms the basis for the activated partial thromboplastin time clotting assay (aPTT). This assay is routinely used to evaluate defects in the intrinsic pathway of coagulation, such as hemophilia A and B. Recent studies have identified endogenous activators of contact system, such as polyphospahte, collagen, misfolded protein aggregates, lipopolysaccharides (LPS), glycosaminoglycans, and nucleic acids [5]. Very recently, we reported that phosphatidylserine may also serve as an activator of contact system in vivo [4].

### Polyphosphate

Polyphosphate was originally identified in nonmammalian cells but was later shown to be enriched in platelet-dense granules [6]. Polyphosphate can be released from activated platelets and can drive contact system activation [6]. Besides platelets, bacteria such as *Escherichia coli*, *Vibrio cholerae*, *Corynebacterium diphtheriae*, *Haemophilus influenzae* contain large amounts of polyphosphate, which is longer than that from platelet and contains > 300 phosphate units [7]. Bacterial polyphosphate activates FXII and initiates bradykinin production that could contribute to leukocyte chemotaxis, pain sensation, and vascular leakage [8].

### Misfolded protein aggregates

Misfolded protein aggregates are a protein activator of the contact system [9]. The in vitro study has shown that a variety of misfolded protein aggregates such as aggregates of amyloid β peptide directly activates FXII, leading to kallikrein activation [9]. Consistently, bradykinin is produced in the cerebrospinal fluid of patients with Alzheimer’s disease. In patients suffering from systemic amyloidosis, a disease in which aggregates of immunoglobulin light chains circulate and deposit, FXII-driven activation of the kallikrein-kinin system is also observed. Binding of FXII to misfolded protein aggregates differs in that to negatively charged surfaces, its binding to surfaces is mediated by the fibronectin type 1 domain, and its binding to aggregates is via the fibronectin type 2, second EGF, and kringle domains.

### Phosphatidylserine (PS)

We recently have reported that in purified systems HK is specifically associated with PS liposome, and is cleaved in the presence of PK and XII [4]. By recognizing PS, HK preferentially binds to apoptotic cells, but not viable cells, which mediates phagocytosis of apoptotic cells (efferocytosis). HK binding to apoptotic cells induces its rapid cleavage to the two-chain form of HK (HKa) and bradykinin. Both the H chain and L chain of HKa are associated with PS liposome and apoptotic cells [4].

### Collagen

Factor XII binds to collagen fibrils of various origins, which are of negative charges [10]. When exposed to plasma, collagen type I induces thrombin formation and plasma clotting, which is dependent on FXII activity [10]. In addition, PK also binds to collagen, thereby inhibiting collagen-induced platelet aggregation [11].

### Cell membrane-mediated assembly and activation of contact system

The physiologic, negatively charged surface for contact system activation is actually the assembly of these proteins on biologic surfaces, ie, cell membranes [1]. The specific interactions with biologic membranes of endothelial cells, platelets, neutrophils, and monocytes indicate that assembly and activation of this system takes place in a physiologic milieu. Contact system proteins can assemble on cell membrane, via binding to their receptors and glycosaminoglycans (GAGs) of proteoglycans including heparan sulphate (HS) and chondroitin sulphate (CS) [12,13]. Detailed investigations of the proteins of the contact system interacting with cells have led to understanding of how this system is physiologically active. The pivotal protein for contact system assembly on cell membranes is HK [14]. HK actually has three domains that fit into the putative kininogen receptor(s) on endothelial cells. Three receptors have been shown to mediate the binding of HK on cell surface, uPAR, gC1qR and cytokeratin-1 [1]. We have reported that HK binds to uPAR via its D5 in membrane rafts of endothelial cells [15], and the D3 and D5 of HK stimulate monocytes via uPAR and Mac1 to produce cytokines and chemokines [16]. Besides healthy cells, apoptotic cells may also provide surface for assemble and activation of contact system [4]. Recently, we have reported that HK bridges uPAR on monocytes/macrophages and phosphatidylserine on apoptotic cells, leading to engulfment of apoptotic cells, and assembly and activation of contact system on apoptotic cell surface [4]. Because efferocytosis is essential for regulation of immune responses and tissue homeostasis, the involvement of HK in recognition and binding of apoptotic cells reveals a novel role of contact system in “apoptotic” innate immunity. Cell surface GAGs expose vast numbers of specific binding sites for contact system. HK and FXII bind with high affinity (K_D_ ≤ 144 nM) to endothelial GAGs in the presence of zinc ions, thereby modulating contact system-driven BK formation [17].

Besides eukaryocytes, prokaryocyes such as bacteria and virus also provide surfaces for assembly and activation of contact system. Contact system proteins bind to both gram negative bacteria such as *Escherichia coli* [18] and gram positive bacteria such as *Streptococcus pyogenes* [19]. Both D3 and D5_H_ of HK are involved in specific binding to bacteria. HK interaction with bacteria resembles protein-binding to endothelial cells, leading to the activation of contact system. Virus, such as herpes simplex virus type-1 (HSV1), is also associated with contact system [20]. HSV1 binds to FXII and mediates its activation in the presence of PK [20].

The activation of contact system triggers both coagulant and inflammatory pathways, and initiates prothrombotic and proinflammatory reactions, via the intrinsic pathway of coagulation (thrombin generation) and the kallikrein-kinin system (kinin production), respectively. As described above, the biochemistry of the contact system in vitro is well understood in the last five decades, recent studies using genetic manipulation in mice, and study of human genetic variability in contact system factors have allowed recognizing the (patho)physiological role of the contact system in health and in disease.

## Contact system in coagulation pathway: activation of the intrinsic pathway

Arterial or venous circulation may undergo thrombosis, causing myocardial infarction and stroke, or pulmonary embolism respectively, the leading cause of death in the world. Coagulation is not only essential to maintain the integrity of a circulatory system (hemostasis), but also contribute to formation of blood clot leading to ischemia and tissue damage (thrombosis). Thrombosis is, triggered by the plasma coagulation system, leading to formation of fibrin. Fibrin formation is initiated by two distinct pathways, through exposure of blood to injured vessel walls (extrinsic) or to blood activation surfaces (intrinsic). When assembled on an activation surface, the contact system (FXII, PK and HK) becomes activated and stimulates the intrinsic pathway of coagulation by activating FXI. In addition, the contact system-mediated intrinsic coagulation pathway may crosstalk with extrinsic coagulation pathway. For example, the contact system of coagulation can be initiated either by the contact surface in plasma from women in late pregnancy or by micellar stearate added to plasma. With either of the contact surfaces, increase of factor VII coagulant activity (VIIc) and FXIIa depended on the potency of the contact surface [21]. The stearate-induced VIIc was inhibited by 60-70% in the presence of anti-factor IX monoclonal antibody and was absent in FXII-deficient plasma. The addition of purified human factor XII to this plasma restored the increase in VIIc. In FIX-deficient or FXI-deficient plasma, the stearate-induced increase in VIIc was greatly reduced, indicating that in the presence of contact surface the activation of contact system results in the activation of FVII [21]. In another in vitro study in which a FXI-dependent effect on clot formation initiated by tissue factor, FXI increased prothrombin activation and the fibrin formation rate, revealing a role for factor XI in the propagation of clot growth after tissue factor-dependent initiation [22].

In contrast to the observations on extrinsic pathway abnormalities, clinical studies showed that human with the deficiency of any contact system factor have no defect in bleeding. For example, Ms. Mayme Williams lacked both forms of kininogen. Like Mr. Hageman (deficient of FXII), she died of a pulmonary embolism [23], demonstrating that patients with contact factor deficiency are not protected against thrombotic disorders. Although the investigation of unique patients with deficiency or contact factors may contribute to our knowledge of their function, contact system deficiency is rare in humans and no controlled large clinical trial has correlated contact system deficiency and pathophysiology such as thrombotic/hemorrhagic disease. Instead, the role of contact system in thrombosis has been recently improved by the studies using animal models. Although the contact system has been considered to have no importance for hemostasis, studies using animal models show that thrombus formation is defective in deficient mice. Thus, the contact system plays a critical role for pathological thrombosis in arterial and venous injury.

### Phenotype of contact factor deficiency in arterial thrombosis

#### FXII deficiency

Renne et al. are the first to report that FXII-deficient mice had defect in thrombus formation induced by different methods of injuries, demonstrating a crucial role of FXII for fibrin formation in vivo [24]. Reconstitution of FXII null mice with human FXII restored the prolonged aPTT found in untreated animals and fully restored the capacity of infused animals for thrombus formation, suggesting the similar role of FXII in mice and humans [24]. Indeed, the contact system is highly conserved among mammalian species. Besides, FXII-gene-deficient mice are protected from cerebral ischemia related to reduced fibrin formation in the microvasculature of the ischemic tissue, consistent with an essential role of FXII for thrombus formation in thromboembolic disease [25]. Mice lacking FXI are similarly protected from vessel occlusive fibrin formation [26]. Thus, the contact system is critical in pathologic clotting via the activation of intrinsic pathway [27]. In contrast to FXII and other contact system proteins, the deficiency of FXI and downstream FVIII and FIX leads to a mild to severe bleeding disorder, suggesting that the intrinsic pathway aids in the amplification of thrombin after initial stimulation of the extrinsic coagulation pathway by tissue factor.

#### PK deficiency

PK is a multifunctional serine protease involved in contact activation of coagulation. Similar to deficiency in humans, PK-deficient mice revealed increased aPTT, without prolonged bleeding time [28]. PK-deficient mice were completely protected from occlusion in FeCl3 -induced arterial thrombosis versus heterozygotes and wild-type littermates [28], demonstrating that plasma prekallikrein deletion prevents occlusive thrombus formation. Consistently, prekallikrein antisense oligonucleotide (ASO) treatment in mice has potential for a positive therapeutic index [29].

#### HK deficiency

HK plays a critical role in the assembly of the contact system. While the human kininogen gene has a single copy, the mouse genome contains 2 homologous kininogen genes, Kng1 and Kng2, which are expressed in a tissue-specific manner [20]. Merkulov et al. found that mKng1 is expressed primarily in the liver, the Kng1-deficient mice lacked plasma HK and low-molecular-weight kininogen (LK), as well as Delta-HK-D5, a kininogen isoform that lacking Kng1 domain 5 [30]. Similar to FXII deficient mice, Kng1^−/−^ mice displayed a significantly prolonged time to carotid artery occlusion following Rose Bengal administration and laser-induced arterial injury [30], suggesting that plasma HK contributes to arterial thrombosis in mice. Kng1^−/−^ mice subjected to transient middle cerebral artery occlusion developed dramatically smaller brain infarctions and less severe neurologic deficits without an increase in infarct-associated hemorrhage [31]. This protective effect was preserved due to reduced thrombus formation in ischemic vessels and improved cerebral blood flow [31]. Since Kng1 deficient mice also showed less severe blood–brain barrier damage and edema formation, and the local inflammatory response was reduced compared with controls, Kng1 appears to be instrumental in pathologic thrombus formation and inflammation but dispensable for hemostasis [31].

When arterial injury occurs, the activated endothelial cells and exposed subendothelial materials such as collagen may serves as surfaces for the assembly and activation of the contact system, leading to FXIa-driven thrombin generation and subsequent platelet activation. Activated platelets may further bind contact system factors such as FXII, mediating platelets-dependent-procoagulant activity [32]. Besides, platelets release FXII-activator polyphosphate, which leads to activation of the contact system and fibrin production on activated platelet surfaces in the developing thrombus. On the other hand, FXIIa has a direct effect on the protease-activated receptor in mice, providing a clue for contact system–driven platelet activation.

### Venous thrombosis

Deep vein thrombosis (DVT) that may cause pulmonary embolism is a life-threatening condition, but its mechanism remains much less understood than arterial thrombosis. Genetic and pharmaceutical studies also demonstrate that the contact system plays a critical role in venous thrombosis. In a mouse FeCl3 injury model, PK-deficient mice exhibit reduced venous thrombosis thrombosis by 50% [28]. Selective knockdown of prekallikrein in mice using antisense oligonucleotide (ASO) technology also results in an antithrombotic phenotype in stenosis-induced inferior vena cava thrombosis model [29]. Similarly, depletion of FXII or XI in rabbits using ASO significantly attenuates catheter-induced venous thrombosis [33]. von Bruhl et al. found that genetic ablation of FXII confers protection against DVT amplification in mice [34]. In their model, thrombus-resident neutrophils binds FXII and supports its activation through the release of neutrophil extracellular traps (NETs) [35], which is indispensable for subsequent DVT propagation [34]. When polyP is intravenously applied, it activates FXII and leads to lethal pulmonary embolism in wild-type mice, which was prevented by either FXII-deficiency or FXII inhibitor, suggesting a critical role of contact system [6,36]. Recombinant Ixodes ricinus contact phase inhibitor (Ir-CPI) is a Kunitz-type protein expressed by the salivary glands of the tick Ixodes ricinus, specifically interacts with activated human FXIIa, FXIa, and kallikrein and prolongs the aPTT in vitro [36]. Intravenous administration of Ir-CPI in rats and mice caused a dose-dependent reduction in venous thrombus formation [36].

Given the results found in mouse models that the mice with deficiency of contact system proteins have normal hemostasis, similar to their human counterparts, the contact system-driven fibrin formation is specifically important for pathologic thrombus formation but has no affection of physiological hemostasis. This raises the possibility that targeting contact system may serve as a strategy for prevention or treatment of pathological thrombosis, which has less risk of severe hemorrhage than currently used anti-coagulants [33]*.* For example, selective depletion of factor XII with antisense oligonucleotides attenuates catheter thrombosis in rabbits [33]. Inhibition of FXII activation using the peptide-based inhibitor PCK in mice also provides protection from cerebral ischemia, without causing excessive bleeding at a surgical injury site. Moreover, a recombinant infestin-4-based inhibitor that specifically targets FXII activity provided protection from cerebral ischemia in experimental stroke models albeit did not affect the hemostatic capacity in inhibitor-treated mice [37].

### Fibrinolysis

Besides the activation of intrinsic coagulation pathway, one of the downstream events of contact system includes fibrinolysis. Activation of plasminogen in vivo can reproducibly be studied by a short lasting infusion of desamino D-arginine vasopressin (DDAVP). Levi et al. found that the formation of plasmin upon the DDAVP stimulus as reflected by circulating plasmin-a2-antiplasmin complexes was lower in FXII-deficient patients than in healthy volunteers, suggesting that activation of the contact system occurred after DDAVP infusion in healthy volunteers [38]. The plasminogen activating activity in normal plasma after infusion of DDAVP was only partially blocked (by two-third) with specific antibodies to tissue-type plasminogen activator (t-PA) and urokinase-type PA. The residual activity could be quenched by an inhibitory monoclonal antibody against FXII and was absent in FXII-deficient patients [38]. Thus, activation of the contact system contributes to fibrinolysis. The impaired fibrinolytic activity in FXII deficient patients may explain the occurrence of thromboembolic complications in these patients. Patients deficient in factor XI suffer from abnormal bleeding. The Ashkenazi Jewish probands with severe FXI deficiency had an increased proportion of episodes of bleeding complications after surgery at sites with enhanced local fibrinolysis, such as the urinary tract, or during tooth extraction, suggesting that FXI plays a role in the downregulation of fibrinolysis [39]. In consistence with this hypothesis, systemic incorporation of anti-factor XI antibodies resulted in an almost twofold increase in endogenous fibrinolysis compared with a control antibody, indicating a novel role for the intrinsic pathway of coagulation [40]. In a static in-vitro coagulation model in which clotting is initiated in recalcified citrated plasma by tissue factor coated on the bottom of microtiter plates, when larger clots (200–300 microl plasma) were formed, FXI not only increased prothrombin activation and the fibrin formation rate but also inhibited fibrinolysis. Thus, in addition to enhancement of tissue factor-initiated coagulation, FXI inhibits fibrinolysis to stabilize the formed clot [22].

## Contact system in inflammation

Contact system is widely involved in inflammatory response and autoimmune disease. It has been known for a long time that the activation of this system produces a potent proinflammatory nona-peptide bradykinin (BK; Arg-Pro-Pro-Gly-Phe-Ser-Pro-Phe-Arg) through cleavage of HK by PK. Thus the contact system was also named plasma kallikrein-kinin system (KKS). Bradykinin and related kinins act on two types of receptors designated as B1R and B2R, both of which are G protein-coupled receptors. B2R is constitutively expressed in various vascular and non-vascular cells and rapidly desensitized. B1R is induced by inflammatory cytokines and resistant to desensitization. These bradykinin receptors are involved in the regulation of various physiological and pathological processes. The mode of kinin actions are based upon the interactions between the kinin and their specific receptors, which can lead to activation of several second-messenger systems. During inflammatory reactions, activated leukocytes and endothelial cells express B1R, which is sensitive to bradykinin-derived peptides. When the kinin B2R is activated, vascular permeability increases, amongst others through NO-induced relaxation of perivascular smooth muscle cells. There is evidence that there is continuous FXII-dependent formation of bradykinin in vivo, as bradykinin levels are reduced by 50% in FXII-deficient mice. BK is rapidly degraded by a number of peptidases, including angiotensin-converting enzyme. As a result, therapeutic targeting of this peptidase elevates BK levels and is being held partially responsible for the blood-lowering effects of this commonly used therapy.

### Infectious disease and sepsis

Vertebrate hosts control infection by rapidly mobilizing soluble and cellular-based microbicidal systems that destroy invading pathogens. Activation of the contact system is a major part of plasma host-defense systems and plays a crucial role as the first line in host defense against pathogens. A growing number of studies indicated that the contact system is important in host defense against infection by bacteria, parasitic protozoan, and virus [20,41,42]. As mentioned above, bacteria may activate the contact system directly through physical interaction, or indirectly by the releases such as proteases and polyP. Similarly to cell surface, contact system proteins bind to bacteria, directly mediating the assembly and activation of the contact system on bacterial surface [43]. Proteinases secreted from bacteria such as staphophains released from Staphylococcus aureus directly cleave HK. Some proteinases such as from Porphyromonas gingivalis indirectly cleave HK by proteolytic activation of FXII or PK [44]. Polyphosphate from bacteria also activates FXII, initiates BK formation and BK-mediated vascular leakage and induces pain sensations. Activation of contact system contributes to host defense against bacterial infection [43]. Both kallikrein and FXIIa activate the complement system, leading to killing of bacteria. Bradykinin may recruitment neutrophil and macrophages to the site of infection, and activate innate sentinel cells via the bradykinin receptor B2R pathway [45]. However, persistent bacteria-driven contact system activation may lead to pathological plasma kinin levels concomitant with consumption of contact factors. In patients, abundant production of BK may induce hypotension and vascular leak, exerting disadvantage effects and contributing to multiple organ failure. Indeed, in severe sepsis patients, the kinin system is activated as exemplified by elevated plasma bradykinin and consumption of prekallikrein and FXII. However, in a double-blind randomized multicenter study, a B2R antagonist showed no improvement in outcome in patients with systemic inflammatory response syndrome and sepsis [46]. This result represents the complexity of the role of contact system in inflammatory cascades.

Activation of contact system is also involved in parasite infection. Trypanosoma cruzi binds HK and cleaves HK by cruzipain, to liberate bradykinin, which is increased up to 35-fold in the presence of heparan sulfate proteoglycans [47]. The liberated kinins activate innate immunity by potently stimulating dendritic cell maturation via the B2R. However, excessive bradykinin leads to persistent parasitism in the edematous tissues. A recent study has shown that activation of contact system is also critical in virus-induced endothelial cell permeability [48]. Hantavirus infection causes vascular leakage due to alterations of the endothelial barrier. Incubation of plasma proteins with hantavirus-infected endothelial cells results in HK cleavage, increase in enzymatic activities of FXIIa and kallikrein and liberation of bradykinin, leading to dramatic increases in endothelial cell permeability [45]. Another virus HSV1 directly binds to FXII and mediates its activation in the presence of PK. An inhibitor of activated FXII, anti-FXII, anti-kallikrein and anti-FXI Abs inhibit HSV1-initiated clotting [20]. Thus virus can trigger and amplify coagulation through the contact phase and intrinsic pathway, suggesting an additional mechanism that contributes to vascular pathology in virus infection.

### Rheumatoid arthritis (RA)

The inflammatory response in arthritis is composed of an acute phase with edema, pain, and neutrophil migration, all of which are known to be associated with plasma kallikrein activation and the release of bradykinin. In patients with RA, elevated levels of plasma kallikrein and bradykinin are detected in synovial fluid and plasma, and positively correlated with the degree of joint pain and inflammation. Bradykinin receptor expression also increased in circulating and synovial neutrophils of RA patients. The concept that the KKS is important in the pathogenesis of arthritis is supported by the observations in animal models. Lewis rats have a mutation in HK (S511N) rendering it susceptible to cleavage by plasma kallikrein, the administration of streptococcal cell wall polymers peptidoglycan - polysaccharide (PG-PS) induces systemic inflammatory response including arthritis in Lewis rats, but not in other strains such as Buffalo and Fischer rats. Accompanied with synovitis and joint erosion in Lewis rats, there is a decrease in plasma prekallikrein and HK, likely due to consumption of the precursor proteins following KKS activation. A specific kallikrein inhibitor prevents arthritis and the systemic complications in the PG-PS model [49]. The Brown-Norway-Katholiek (B/N/Ka) rat strain has a severe deficiency of both plasma kininogens (HK and LK) due to a single point mutation, Ala163Thr, which results in defective secretion of kininiogen from the liver. Kininogen-deficient rats on a Lewis genetic background exhibit attenuated acute and chronic inflammatory arthritis [50], demonstrating that plasma HK is a key mediator of inflammatory diseases.

Although these experimental and clinical observations demonstrate that the KKS plays a critical role in the pathogenesis of arthritis, the molecular and cellular mechanisms by which the activation of the KKS mediates arthritis remains unknown. Recently we report that the inhibition of plasma kallikrein ameliorates arthritis in two Lewis rat models, PG-PS model and collagen-induced arthritis model, the effect of plasma kallikrein is probably through bradykinin [51]. Bradykinin has two receptors, constitutively expressed B2R and inducible B1R both belong to G protein-coupled receptors. A recent study by Song et al. demonstrated B2R deficiency did not attenuate arthritis in a mouse model of anti-collagen antibody-induced arthritis (CAIA) [52]. However, using the same CAIA model, we found that the double deficiency of B1R and B2R significantly inhibits the development and severity of CAIA [53]. A decrease in plasma HK level was observed at early stage of CAIA [53], suggesting the activation of the KKS and generation of bradykinin at acute phase of the disease. These observations provide the first genetic evidence showing that bradykinin is critical in the pathogenesis of CAIA and B1R is likely a key receptor that mediates the proinflammatory effect of bradykinin in arthritis.

The function of bradykinin receptors in arthritis seems universal, including local joint tissue and circulating inflammatory cells. First, the KKS components can enter synovial joint space either by transudation from the plasma or from degranulating neutrophils chemotactically attracted into the synovium. Excessive release of kinins in the synovial tissues may stimulate the synthesis of second wave of pro-inflammatory mediators (IL-1β,IL-6, prostaglandins, leukotrienes, and histamine) to enhance inflammatory joint disease. Upregulated expression of bradykinin receptors in joints may increase cytokine production. Second, upregulated bradykinin receptor expression in circulating monocytes may enhance systemic inflammation and their homing to inflamed synovial tissue. The expression of B1R in CAIA can be induced by proinflammatory cytokines such as IL1β, IL-6, and likely occurs as part of an proinflammatory feedback mechanism. B1RB2R deficiency significantly inhibited IL-1β and IL-6 mRNA expression, but not that of TNFα mRNA, suggesting that bradykinin receptors differently regulate these cytokine expressions, and their role in the pathogenesis of CAIA is, at least in part, through the regulation of IL-1β and IL-6.

Clinical and experimental observations have suggested that synovial neovascularization is a critical component of disease progression in RA [54,55]. There is a growing body of evidence indicating that bone marrow-derived endothelial progenitor cells (EPCs) participate in synovial neovascularization in arthritis [56,57]. Thus, how EPCs are recruited to the inflamed synovium and participates in synovial neovascularization becomes an important issue. Recently, we have demonstrated that HKa regulates EPC functions in vitro [58,59], revealing the connection between the KKS activation and EPC biology. In rodent model of arthritis, we found another role of the KKS in the homing function of EPCs in arthritis [51]. In Lewis rat model of arthritis, EPCs are recruited to inflamed synovium and form new vessels, which was blocked by specific plasma kallikrein inhibitor; Bradykinin stimulates transendothelial migration of EPCs by selectively upregulating expression of homing receptor C-X-C motif receptor 4 (CXCR4) [51]. Our study provides novel evidence that activation of the KKS is associated with synovial recruitment of EPCs at acute phase of arthritis, EPCs are likely a new target for bradykinin in the setting of acute arthritis. The multiple function of contact system in arthritis demonstrates that therapeutic approaches targeting the KKS activation have considerable potential in arthritis.

### Inflammatory bowel disease (IBD)

IBD is a group of inflammatory conditions of the colon and small intestine, its principal types include ulcerative colitis (UC) and Crohn’s disease (CD). Plasma KKS activation has been observed in patients with IBD [60]. In active ulcerative colitis patients, a significant decrease of plasma prekallikrein, high molecular weight kininogen, and C1 inhibitor levels was observed as compared with controls. The decreases in KKS components results from the consumption by activation. Activation of the KKS may mediate inflammation in the active phase of ulcerative colitis. In Crohn’s disease patients, plasma levels of prekallikrein, factor XI, HK and its cleaved form were normal. However, they had significantly higher levels of antigen and functional Cl-inhibitor. The undetectable contact system activation in peripheral blood might be related to the high plasma levels of Cl-inhibitor, an important inhibitor of the contact system in the circulation [61]. Both B2R and B1R proteins are expressed in the epithelial cells of normal intestines and those from patients with UC and CD. B1R protein is localized in macrophages at the center of granulomas in CD. B2R protein expression in the apexes of enterocytes in the basal area and intracellularly in inflammatory tissue is similar with normal tissue. In contrast, B1R protein is localized in the basal area of enterocytes in normal intestine but in the apical portion of enterocytes in inflamed tissue. Compared with normal controls, B1R protein is significantly increased in both active UC and CD intestines [61]. In patients with active UC, B1R mRNA is significantly higher than B2R mRNA. However, in inactive UC patients, the B1R and B2R mRNA are comparably expressed. Thus, increased B1R gene and protein expression in active IBD provides a structural basis of the important role of bradykinin in chronic inflammation, and may reflect intestinal inflammation.

The role of KKS in IBD has been revealed by studies using kallikrein inhibitors, bradykinin antagonists or kininogen deficiency. Kinetics of inflammation in inbred Lewis rats injected with PG-PS is correlated with activation of the contact system. Lewis rats had a biphasic course of enterocolitis. Consumption of the precursor proteins PK and HK in Lewis rats indicates activation of the plasma contact system and closely correlated with chronic intestinal inflammation. In another indomethacin induced enterocolitis model, rat displayed KKS activation manifested by a significant decrease in plasma prekallikrein and HK functional levels, and by HK cleavage [62]. Since KKS activation occurs in association with intestinal injury, regardless of the triggering agent, suggesting that activation of this system is integrally involved in intestinal inflammation in genetically susceptible hosts. Inhibition of plasma kallikrein significantly decreased acute intestinal inflammation and more dramatically reduced the tissue granulocyte recruitment. Moreover, in PG-PS model of IBD, HK deficiency modulates chronic intestinal inflammation [63]. These results indicate the importance of the contact system in chronic enterocolitis and systemic inflammation.

Recent studies using mouse model of IBD have shed light onto the mechanism underlying the role of contact system in the IBD pathogenesis. Colitis induced by 2,4,6-trinitrobenzene sulphonic acid (TNBS) is associated with tissue damage, neutrophil infiltration, which can be reduced by the selective B1R antagonist SSR240612 and B1R deficiency [64]. Although these results suggest the role of B1R in the pathogenesis of IBD, this effect seems model-specific. In dextran sulfate sodium (DSS)-induced colitis model, colitis was significantly exacerbated in B1R^−/−^ mice compared with wild-type mice. Moreover, treatment with a selective B1R antagonist, DALBK or SSR240612, had no effect on DSS-induced colitis [64]. In contrast, a selective B2R antagonist prevented the exacerbation of colitis in B1R^−/−^ mice. B2R mRNA expression was significantly upregulated in colonic tissue from the B1R^−/−^ mice after DSS administration. Thus, exacerbation of DSS-induced colitis in mice lacking B1R is through compensatory up-regulation of B2R. Although this study suggests that B2R is a major receptor mediating the role of bradykinin in IBD, B2R^−/−^ mice is not protected from the disease in the same DSS model [65]. Thus, the role of bradykinin and specific involvement of its two receptors in the pathogenesis of IBD is very complex, more work is warranted to evaluate their dynamic cooperation and distinct involvement in the development and progression of the diseases.

Thrombosis and inflammation are hallmarks of a variety of tissue injury unamenable to therapeutic interventions. Since contact system plays an important role in both processes, it represents an interface in multiple ways between thrombotic and inflammatory circuits and is a critical mediator of tissue injury development. The specific role of the contact system in both processes and their crosstalk requires further investigations.
